# A cross-sectional study of vitamin D levels in a large cohort of patients with rheumatic diseases

**DOI:** 10.1007/s10067-017-3870-8

**Published:** 2017-11-07

**Authors:** Elena Nikiphorou, Jaakko Uksila, Tuulikki Sokka

**Affiliations:** 1 Rheumatology Department, Jyvaskylä Central Hospital, Jyväskylä, Finland; 2 Academic Rheumatology Department, King’s College London, London, UK; 3 Laboratory, Jyvaskylä Central Hospital, Jyväskylä, Finland; 4 Faculty of Health Sciences, University of Eastern Finland, Jyväskylä, Finland

**Keywords:** 25-Hydroxyvitamin D, Autoimmune disease, Patient-reported outcomes, Rheumatoid arthritis, Vitamin D-25 deficiency

## Abstract

The objective of this study is to examine 25-hydroxyvitamin D [25(OH)D] (D-25) levels and associations with patient- and disease-related factors in rheumatic diseases. This is a register-based study of D-25 levels in adult patients seen at the Central Finland Hospital rheumatology clinic (January 2011–April 2015). Demographic, clinical, laboratory, and patient-reported outcomes (PROs) were collected as part of the normal infrastructure of the outpatient clinic and examined for their association with D-25 level. Statistical analysis included descriptive statistics and univariable and multivariable regression analyses adjusting for age and gender. D-25 was measured in 3203 patients (age range 15–91 years, mean 54; 68% female) with diagnoses including RA (*n* = 1386), unspecified arthralgia/myalgia (*n* = 413), and connective tissues diseases (*n* = 213). The overall D-25 mean (SD) level was 78 (31) and median (IQR) 75 (55, 97). At baseline, 17.8% had D-25 deficiency, and only 1.6% severe deficiency  (< 25 nmol/l); 34%/49% had sufficient/optimal D-25 levels. Higher D-25 levels were associated with older age, lower BMI, and regular exercise (all *p* < 0.001) among other factors. In multivariable analyses, younger age, non-white background, higher BMI, smoking, less frequent exercise (*p* < 0.001), and first visit to the clinic (*p* = 0.033) remained significantly associated with D-25 deficiency. Among those with sub-optimal D-25 levels, 64% had improved to sufficient/optimal levels after a median (IQR) of 13 (7.8, 22) months. The proportion of patients with D-25 deficiency in this study was generally low. Older patients had considerably higher D-25 levels compared to younger patients. Lower physical exercise and higher BMI were associated with higher risk of deficiency. The study supports the benefit of strategies to help minimize the risk of D-25 deficiency.

## Introduction

Vitamin D (D-25) deficiency has represented a growing concern over the years, with a recognized higher risk at northern latitudes and in winter and spring seasons [1, 2]. Reports on the re-emergence of rickets in Europe in the twenty-first century [3–6] have further strengthened concerns around the persistence of D-25 deficiency states and resulted in a number of mandatory food fortification and specific supplementation programs in various countries at risk. Epidemiological data suggest that D-25 deficiency may be a risk for the development of autoimmune, chronic diseases [2, 7] including RA [8–10]. A meta-analysis showed a small reduction in all-cause mortality after D-25 supplementation among older adults [11]. D-25 deficiency has been associated with non-specific musculoskeletal pain in acute rehabilitation unit patients [12].

Despite evidence supporting the pronounced effects of D-25 deficiency in patients with known chronic musculoskeletal conditions, it does not always represent a priority treatment at all rheumatology clinics. There have been few studies examining the relationship between D-25 levels and RA disease outcomes such as functional status, physical exercise, and work status. A previous study [8] reported low D-25 levels to be an independent predictor of greater disability in patients with RA, but patient numbers were small and based on academic-setting rheumatology clinics.

The growing concerns around D-25 deficiency and impact on outcomes provided the rationale for this study. The objective was to analyze D-25 levels (25-hydroxyvitamin D [25(OH)D]) and its associations with patient and disease outcomes (clinical, laboratory, and patient-reported) using routine clinical care data at a large rheumatology outpatient clinic in central Finland.

## Materials and methods

### Study design and population

This was a register-based, cross-sectional study which examined data from 3202 adult patients seen at the rheumatology clinic at Jyväskylä Central Hospital, Finland, between January 2011 and April 2015. Jyväskylä Central Hospital is the only rheumatology specialty unit in the Central Finland Health Care District serving a population of over a quarter of a million (5% of the Finnish population) in Central Finland District, which is located between the latitudes of 61 and 63 N. All new adult patients with suspected rheumatic diseases are referred to this center for diagnosis and management and those confirmed with rheumatic disease have regular follow-ups in outpatient and day-care units [13]. For over 20 years and throughout the period of this study, calcium and D-25 supplementation has been advised and prescribed routinely at the first clinic appointment, especially as many of these patients will be prescribed glucocorticoid treatment. This has been the policy of our clinic even before vitamin D testing was undertaken. If low vitamin D was identified in these patients, they were instructed to take additional supplementation. Furthermore, in Finland, an active national health promotion program regarding D-25 supplementation and fortification of milk and dairy products [14] further increases general public awareness. Measurement of D-25 levels has not been a routine practice in health care or in rheumatology clinics until it was implemented gradually since January 2011 at the rheumatology unit.

### Data source

Patients had their demographic, laboratory, clinical, and self-reported data collected as part of the normal infrastructure of the outpatient clinic using the electronic monitoring tool GoTreatIT [15]. Variables examined for are shown in Table 1.

**Table 1:** Description of variables extracted from GoTreatIT monitoring system and used in the analysis

Variable	Description
Demographic features	
Age	Age at disease onset
Gender	Male vs female
Ethnic origin	White vs non-white
Body mass index	Self-reported weight in kilograms divided by the square of height in meters
Smoking	Current smoking vs non-smoking
Education	Length of education in years
Disease characteristics	
Diagnosis	Formal rheumatological diagnosis given by physician
Disease duration	Calculated from clinical diagnosis to visit date (in years)
Patient-reported outcomes	
HAQ	Health Assessment Questionnaire, range 0–3; higher scores imply more disability
Pain	Pain on 0–100 mm Visual Analogue Scale (VAS)
Patient global	Patient assessment of global health on 0–100 mm VAS
Patient disease activity	Patient assessment of disease activity on 0–100 mm VAS
Early morning stiffness	Recorded in minutes
Fatigue	Fatigue on 0–100 VAS
Physical exercise	Self-reported engagement in physical activity (>half hour/day with sweating, increased frequency in breathing) categorized as follows: > = 3 times/week; 1–2 times/week; 1–2 times/month; none—categorized for analyses as: regular exercise > = 3 times/week vs irregular/no exercise
Laboratory variables	
ESR	Erythrocyte sedimentation rate
25-Hydroxyvitamin D [25(OH)D]	Serum vitamin D level^a^
Crea	Creatinine
Hb	Hemoglobin
ALT	Alanine transaminase

^a^See separate section in main document

### Serum vitamin D [25(OH)D] measurements

Through the observation period, serum 25(OH)D concentrations were determined using automated competitive electrochemiluminescence binding assay (ref. 05894913 190, Modular Analytics E170 analyzer; Roche) according to the manufacturer’s instructions. Cutoff levels were defined in accordance with the Finnish Current Care Guidelines [16, 17]: < 25 nmol/l = severe deficiency; < 50 nmol/l = deficiency; 50–75 nmol/l = sufficient; > 75 = optimal; > 375 = toxic.

### Statistical analysis

Descriptive statistics including mean, standard deviation (SD), 95% confidence intervals (95% CI), median, and inter-quartile range (IQR) were used in the descriptive part of the analysis. Differences between groups were analyzed using the Student’s *t* test for continuous and chi-square test for categorical variables. Univariable and multivariable (adjusting for age and gender) regression analyses were used with D-25 examined as both a continuous and categorical variable, based on cutoffs indicating specific D-25 status (see above).

## Results

### Patient characteristics

A total of 3203 patients seen at Jyvaskyla Central Hospital rheumatology clinic between January 2011 and April 2015 had their D-25 levels measured. Patient age ranged from 15 to 91 years, mean age 54; 68% were female; 8.4% of the patient population was 15 to 25 years old (4.1% 15 to 20 years). All but 29 patients were North European origin. The majority of the patients (43%) had rheumatoid arthritis (RA), non-specific arthralgia/myalgia (13%), and undifferentiated arthritis (6.7%). See Table 2 for patient characteristics.

**Table 2:** D-25 levels (nmol/l) by diagnosis group and patient characteristics

Diagnosis group	Total *n*	Mean age	Female (%)	Mean (SD) D-25 level	Median (IQR) D-25 level	% less than 50 nmol/l
Rheumatoid arthritis	1386	61	70	81 (31)	78 (58, 101)	15.4
Arthralgia/myalgia (non-specific)	413	44	74	71 (27)	71 (50, 87)	23.7
Undifferentiated arthritis	216	47	63	74 (28)	72 (53, 91)	19.4
Ankylosing spondylitis (axial)	192	44	43	76 (34)	71 (55, 93)	19.3
Vasculitis	164	66	65	86 (30)	82 (62, 108)	9.1
Psoriatic arthritis	138	52	40	72 (28)	70 (52, 90)	21.0
Juvenile idiopathic arthritis	122	31	71	74 (31)	68 (50, 99)	23.8
Connective tissue diseases	213	53	85	82 (32)	80 (60, 98)	14.6
Osteoarthritis	96	64	83	76 (29)	76 (55, 93)	18.8
Fibromyalgia	67	49	87	74 (32)	67 (50, 90)	23.9
Crystal arthritis	36	62	22	74 (34)	69 (57, 83)	16.7
Back pain	33	40	73	77 (31)	78 (50, 98)	21.2
Sarcoidosis	10	54	70	70 (21)	68 (50, 85)	20
Unknown	117	50	72	74 (31)	69 (51, 94)	21.4
All patients	3203	54	68	78 (31)	75 (55, 97)	17.7

### D-25 levels by diagnosis group

**Fig. 1:**
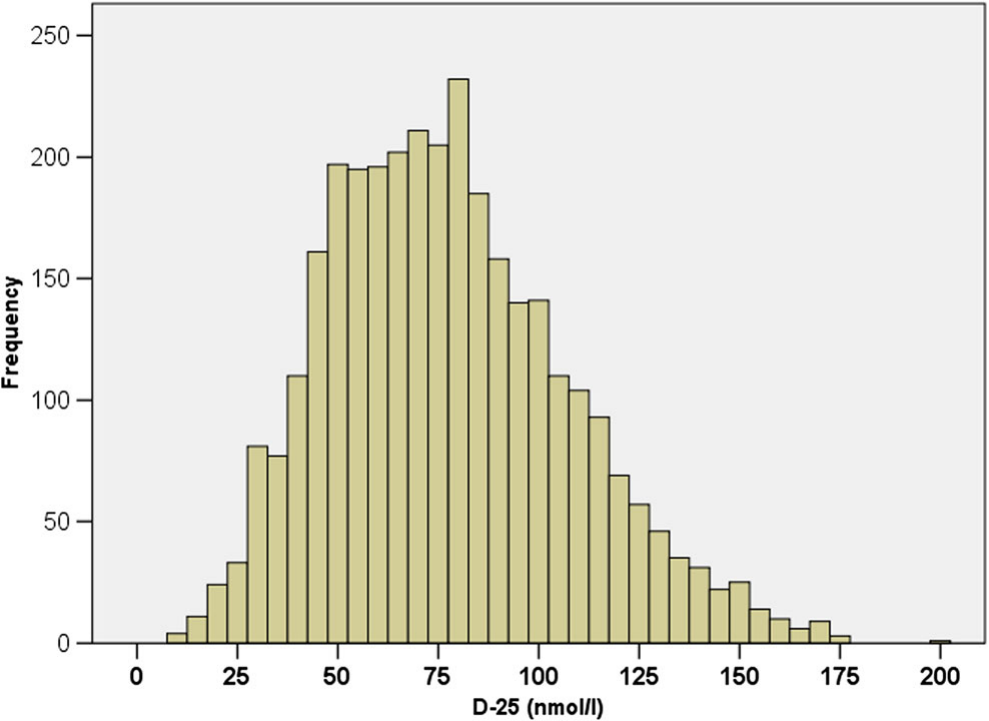
Baseline vitamin D levels in 3203 patients in a rheumatology clinic

**Fig. 2:**
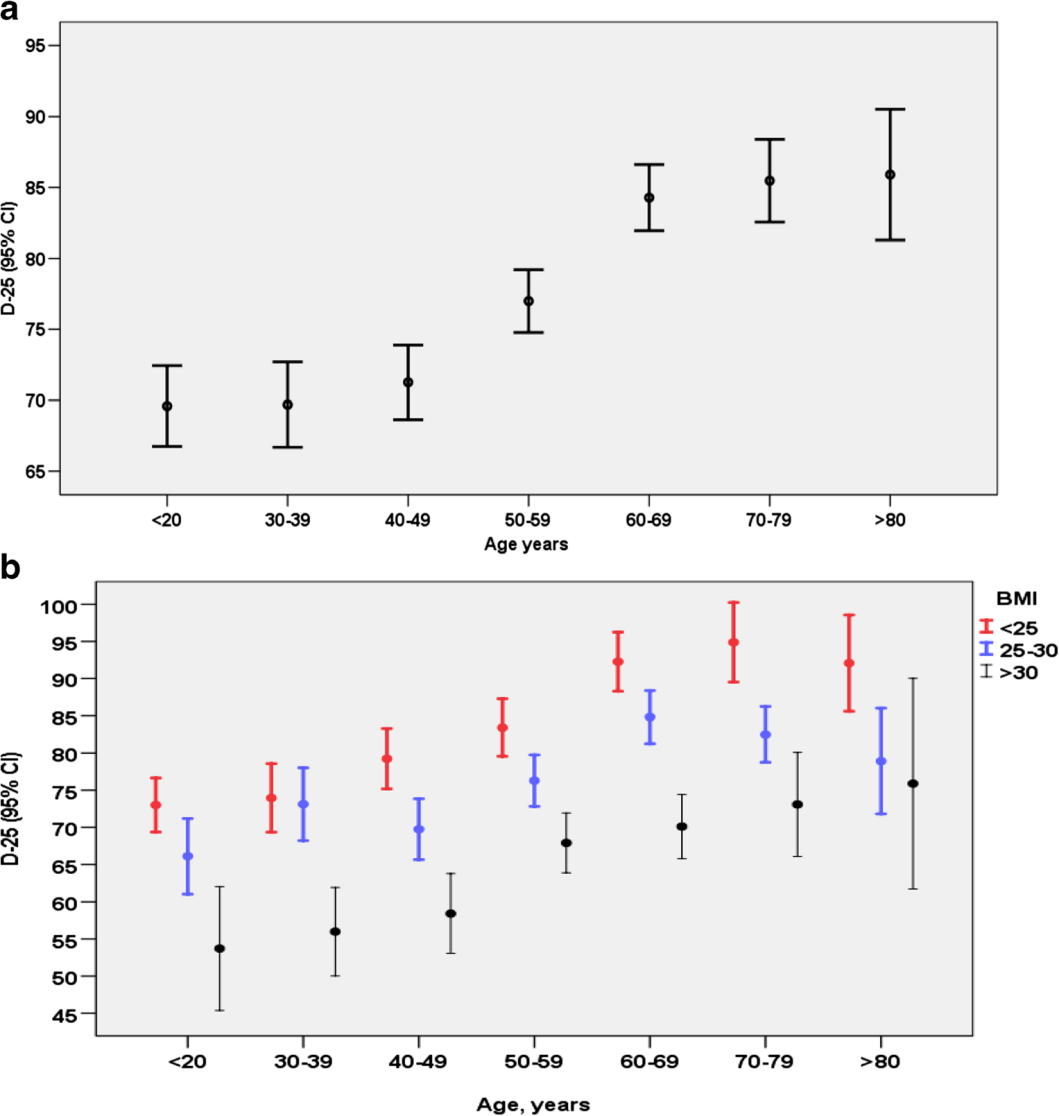
**a**, **b** Vitamin D levels in 3203 patients in a rheumatology clinic, by age (**a**), age and BMI (**b**)

The overall mean (SD) level of D-25 was 78 (31) nmol/l, and median (IQR) 75 (55, 97). Overall, 17.8% had D-25 deficiency of < 50 nmol/l, with only a very small proportion (*n* = 52; 1.6%) having severe deficiency of < 25nmol/l (Fig. 1). The majority (*n* = 1589; 49.6%) had optimal levels (D-25 > 75 nmol/l) and 32.7% (*n* = 1047) sufficient D-25 levels of 50–70 nmol/l. Vitamin D levels by age and BMI are shown in Fig. 2. No patient had toxic levels of D-25. Table 2 summarizes D-25 levels and patient characteristics by diagnosis group. Overall, mean/median D-25 levels were higher in patients with vasculitis or connective tissue diseases (CTDs).

The highest proportion of patients with deficient D-25 levels was seen in the fibromyalgia, juvenile idiopathic arthritis (JIA), and non-specific arthralgia/myalgia diagnosis groups despite mean D-25 levels greater than 70 nmol/l in all these groups.

### Association between D-25 levels and patient characteristics

D-25 levels were higher in patients seen at a follow-up visit in the clinic compared to patients who were seen at their first visit. Mean (95% CI) D-25 levels were 80.8 (79.3 to 82.3) and 74.3 (72.8 to 75.8), (*p* < 0.001); respectively. Mean (95% CI) D-25 levels were higher in women compared to men: 78.6 (77.3 to 79.8) vs 76.2 (74.3 to 78.1); (*p* = 0.044), in non-smokers vs smokers: 78.6 (77.4 to 79.8) vs 73.1 (70.3 to 75.8); (*p* < 0.001), and in patients who reported regular physical exercise vs non-regular exercise: 82.0 (80.2 to 83.8) vs 75.3 (74.0 to 76.6); (*p* < 0.001). Length of education did not have a significant association with vitamin D-25 levels.

**Fig. 3:**
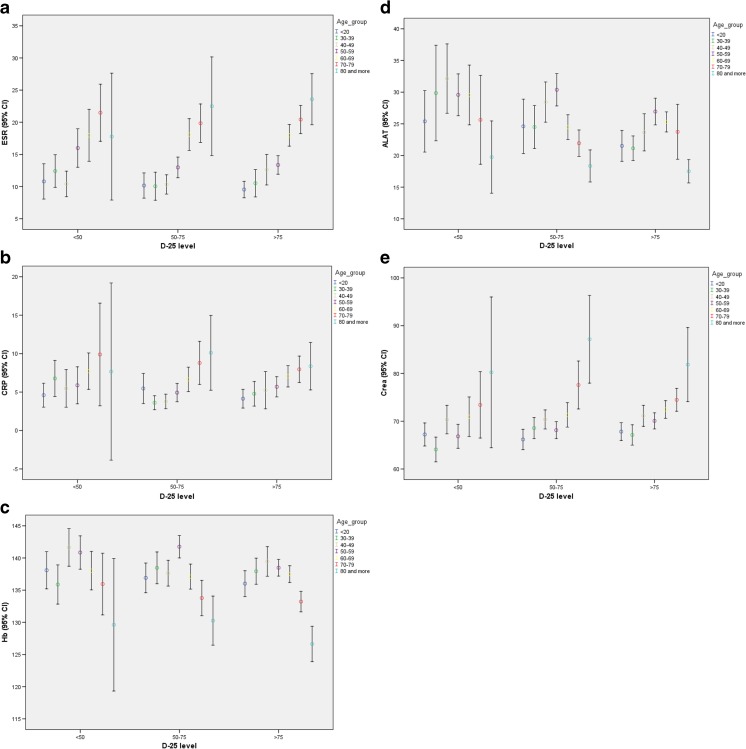
**a**–**e** Laboratory measures in relation to vitamin D-25 level and age

D-25 levels increased by age (Fig. 3a). Lower BMI was associated with higher D-25 levels across all age groups (Fig. 3b).

Among 29 patients with darker skin, 20 (69%) had D-25 deficiency, while the equivalent figure was 17% among 3174 patients with Northern European origin.

### Correlation between D-25 level, laboratory tests, and PROs

Age rather than the D-25 level was associated with ESR, CRP, hemoglobin, ALT, and serum creatinine levels (Fig. 3a–e).

None of the patient-reported outcomes (PROs) correlated significantly with D-25 levels such as HAQ, pain, patient global score, and fatigue (data not shown).

### Multivariable analysis of associates of low D-25

In multivariable analysis, younger age (*p* < 0.001), higher BMI (*p* < 0.001), and lack of regular exercise (*p* = 0.001) remained significant for the risk of D-25 deficiency < 50. Patients of non-white background were 7.5 times (up to 17 times) more likely to be D-25 deficient compared to those of white background (*p* < 0.001). Smoking was associated with higher risk of D-25 deficiency (*p* = 0.001). Also, first visit to the clinic (*p* = 0.033) remained significant for the risk of D-25 deficiency (Table 3).

**Table 3:** Multivariable analysis model of factors associated with low D-25

	*B* coeff	*p* value	Exp (*B*)	95% CI for exp. (*B*)	
				Lower	Upper
Sex, female vs male	− 0.083	0.428	0.920	0.750	1.130
Age	− 0.026	< 0.001	0.974	0.969	0.980
Race, non-white vs white	2.015	< 0.001	7.503	3.298	17.067
Smoking now vs non-smoking	0.405	0.001	1.499	1.171	1.919
BMI	0.072	< 0.001	1.075	1.057	1.093
Physical exercise, regular vs not-regular	− 0.353	0.001	0.703	0.569	0.869
Visit, return visit to clinic vs first	− 0.213	0.033	0.808	0.665	0.983
Constant	− 2.001	< 0.001	0.135		

### Vitamin D-25 levels at follow-up

Roughly one third of the patients had D-25 re-measured after a median (IQR) of 13 (7.8, 22) months. Among those who had sub-optimal values for D-25, 64% had improved to sufficient or optimal levels (data not shown).

## Discussion

This cross-sectional study of D-25 levels in patients with rheumatic diseases in central Finland indicates that the great majority had optimal levels. This is particularly relevant in current times as there have been several reports to date demonstrating lower D-25 levels in Nordic countries and increasing prevalence of rickets, cancer, diabetes, obesity, and morbidities may be associated with D-25 inadequacy [17]. In our study, we observed an increasing D-25 level with increasing age; in other words, older patients had higher D-25 levels than younger patients. This is a paradox considering that with older age, one would expect lower D-25 intake, intestinal absorption, and capacity to convert vitamin D to its active form in the kidneys and skin. A possible explanation for this observation could be precisely the active D-25 screening and supplementation strategies employed both at the clinic but also at national level, minimizing the risk of D-25 deficiency.

Only 17.8% of patients had D-25 deficiency, and in only less than 2% this was severe. Based on D-25 levels by diagnosis group, mean levels ranged between 70 and 86 (sufficient/optimal range). Patients with vasculitis and CTDs were found to have the highest levels of mean and median D-25 levels across all diagnosis groups, which could reflect more targeted supplementation in patients at risk or with known autoimmune diseases such as systemic lupus erythematosus and with potentially higher glucocorticoid requirements [18, 19].

The role of D-25 in calcium homeostasis, bone mineralization, and preservation of bone health is well established, as is the need for adequate D-25 levels for general musculoskeletal health. Therefore, patients with rheumatic diseases represent a high-risk group. The analysis of D-25 levels by diagnosis group in the study could help in the identification of vulnerable patient groups that should be more closely targeted. Diagnosis groups with the highest proportion (around 1 in 5) of patients having deficiency were the JIA, fibromyalgia, and the non-specific arthralgia/myalgia groups. For the latter two, lower D-25 levels could have a causal role and could reflect a particularly vulnerable group of patients who should be more closely targeted. With regard to JIA patients, one could speculate that their younger age (mean age 31) could predispose to lower health awareness and less engagement in healthy lifestyle, dietary habits, and supplementation intake despite recommendations. This finding is supported by the multivariable regression analysis where younger age was a significant independent predictor of D-25 deficiency. However, only 8.4% of the patient population was below the age of 25; the prevalence of D-25 deficiency may be much higher in young people in the general population.

The study identified non-white ethnic background, less physical exercise, smoking, and higher BMI as further independent predictors of D-25 deficiency, suggesting a possible target group of patients. These variables could represent surrogates of a healthy lifestyle. Women were also found to have higher levels of D-25 compared to men, supporting previous data [20]. This could suggest gender-related pathophysiological mechanisms at play, or simply differences in health and dietary perceptions and supplement intake.

The finding of generally higher D-25 levels in this study contradicts findings from previous studies where D-25 deficiency, especially in countries at northern latitudes, has been a major problem [2–6]. We can speculate that the local clinic policy on the background of mandatory food fortification (e.g., of liquid milk and dairy products) and appropriate supplementation programs along with health campaigns since the early 2000s [14] are at least partly accountable for the results. Improvements in D-25 states at a national level in Finland have been suggested in the latest dietary survey FINDIET in 2012 [21], where Finnish adults met the D-25 recommendations on average, except in older women. The example of Finland raises hope that such programs and interventions at a national level could positively influence population outcomes, as seen with the example of D-25.

The finding of lower education correlating significantly with higher D-25 levels was interesting as one would expect the two to be in the same direction. However, this could in fact highlight that despite lower individual education levels, nationwide health promotion programs and local supplementation policies can be equally effective for this purpose. Another explanation could be that patients with lower education may have more manual-labor occupations and hence more time spent outdoors and higher sunlight exposure. The study showed that in patients whose care was maintained in the clinic as follow-up visits, compared to first time visitors, had higher D-25 levels and this could indirectly suggest more opportunity for education.

The large patient numbers and examination of demographic, clinical, and patient-reported variables common across several disease groups represent strengths of the study. Limitations include its observational, cross-sectional nature which makes associations between variables challenging to interpret. The cross-sectional analysis meant that not all D-25 measurements were at baseline (i.e., first patient review in clinic) but some were at different time points, which is a further limitation of the study. However, this way it was possible to examine for differences in D-25 levels based on follow-up time. Furthermore, the examination of associations between D-25 levels and specific outcomes at single time points is a possible source of bias and could have prevented real effects and associations from being seen.

This study provides evidence that D-25 national policies and health promotion campaigns along with local/departmental polices for the most vulnerable population groups can be effective. Such strategies may be more effective in some populations compared to other and it is important that national, cultural, and dietary habits are taken into account [14]. The study raises hope on a health issue that has represented a growing concern over time, demonstrating that D-25 deficiency states can be actively prevented. We further conclude that there is value in testing D-25 levels in patients with rheumatic diseases and especially those at high risk.
